# Cost-effectiveness of conservative treatments for neck pain: a systematic review on economic evaluations

**DOI:** 10.1007/s00586-012-2272-5

**Published:** 2012-03-25

**Authors:** Maurice T. Driessen, Chung-Wei C. Lin, Maurits W. van Tulder

**Affiliations:** 1 Department of Health Sciences, The EMGO Institute for Health and Care Research, Faculty of Earth and Life Sciences, VU University Amsterdam, Amsterdam, The Netherlands; 2 The George Institute for Global Health and Sydney Medical School, The University of Sydney, Sydney, Australia; 3 Department of Health Economics and Health Technology Assessment, Faculty of Earth and Life Sciences, de Boelelaan 1085, 1081 HV Amsterdam, The Netherlands

**Keywords:** Systematic review, Cost-effectiveness, Neck pain, Conservative treatment, Economic evaluation

## Abstract

**Purpose:**

Various conservative interventions have been used for the treatment of non-specific neck pain. The aim of this systematic review was to investigate the cost-effectiveness of conservative treatments for non-specific neck pain.

**Methods:**

Clinical and economic electronic databases, reference lists and authors’ databases were searched up to 13 January 2011. Two reviewers independently selected studies for inclusion, performed the risk of bias assessment and data extraction.

**Results:**

A total of five economic evaluations met the inclusion criteria. All studies were conducted alongside randomised controlled trials and included a cost-utility analysis, and four studies also conducted a cost-effectiveness analysis. Most often, the economic evaluation was conducted from a societal or a health-care perspective. One study found that manual therapy was dominant over physiotherapy and general practitioner care, whilst behavioural graded activity was not cost-effective compared to manual therapy. The combination of advice and exercise with manual therapy was not cost-effective compared to advice and exercise only. One study found that acupuncture was cost-effective compared to a delayed acupuncture intervention, and another study found no differences on cost-effectiveness between a brief physiotherapy intervention compared to usual physiotherapy. Pooling of the data was not possible as heterogeneity existed between the studies on participants, interventions, controls, outcomes, follow-up duration and context related socio-political differences.

**Conclusion:**

At present, the limited number of studies and the heterogeneity between studies warrant no definite conclusions on the cost-effectiveness of conservative treatments for non-specific neck pain.

## Introduction

Non-specific neck pain is a common condition amongst the general population. Prevalence rates show that up to almost two of every three persons will experience neck pain at a certain time during their life. One-year prevalence rates for neck pain range between 20 and 40 % [1, 2]. Neck pain is a financial burden for society, since these symptoms result in extended periods of sick-leave from work and high utilisation of health care services. In the Netherlands, the total health care costs in 1996 for the treatment of neck pain are estimated at €485 million [3]. Considering the rising costs of health care, it is plausible that these estimates would be higher today. Numbers obtained from the United States (US) showed that in the period from 1997 to 2006, the US health care expenditures have increased 7 % per year for persons with spinal problems [4]. In 2005, spinal problems accounted for 9 % of the total US health care expenditures [5].

Economic evaluations investigate the value for money of health care interventions. The costs and effects of the health care intervention under study are compared with the costs and effects of an alternative intervention. This comparison gives insight into whether a health care intervention is worth implementing. For policy makers, health care professionals, and patients, this information is important to decide whether or not to reimburse, provide or receive a specific intervention.

Various conservative treatments are applied as treatment for non-specific neck pain, including guideline care by the general practitioner, manual therapy, physiotherapy, graded activity programmes, and combinations of these treatments. The results obtained from studies on the effectiveness of conservative neck-pain treatments have been already summarised in systematic reviews [6, 7]. High-quality evidence showed that the combination of manual therapy and exercise therapy was more effective to reduce the pain intensity at the short-term among (sub)acute and chronic neck-pain patients when compared with only exercise therapy. Amongst chronic neck-pain patients, moderate-quality evidence showed that this treatment combination in comparison to manual therapy was more effective to reduce pain intensity and to improve quality of life. However, none of these recent reviews provided information on the cost-effectiveness of these treatments for neck pain. For this reason, we conducted a systematic review.

## Methods

### Search strategy

The electronic databases Medline, EMBASE, EconLit, EURONHEED, and NHSEED were searched from inception to 13 January 2011 (refer for example of Medline search to Appendix). Additional articles were identified from reference lists of systematic reviews and key publications on non-specific neck pain, and the authors’ own literature databases.

### Study selection

Two reviewers independently (MTD, CCL) screened the obtained titles and abstracts on the eligibility. Studies were eligible when all three inclusion criteria were met: (1) the study encompassed a full economic evaluation (i.e., cost-minimisation, cost-effectiveness, cost-utility or cost-benefit analysis) comparing costs and effects of at least two interventions from any perspective was included; (2) the study included patients with non-specific neck pain, indicating that the neck pain is not caused by a trauma or an accident and is not classified as specific neck pain (i.e., tumour, fracture, hernia nuclei pulposi, spondylolisthesis, inflammation or infection); (3) the study reported on both the costs and effects or provided an incremental cost-effectiveness ratio (ICER). Studies were excluded when: (1) the study collected data on costs and/or utilisation but did not relate this information to a measure of benefit, or did not make inferences about the relative efficiency of the treatment alternatives; (2) the study reported on multiple musculoskeletal disorders (e.g., neck, shoulder, arm, and/or low back) but did not separately present the costs and effects for neck pain; (3) the study was not written in the English language.

When inclusion or exclusion of a study could not be based on the screening of the title and abstract, the full article was retrieved and checked for inclusion. A consensus meeting with a third reviewer (MvT) was arranged if disagreements between the two reviewers persisted.

### Risk of bias assessment

Using the 19 criteria Consensus Health Economy Criteria (CHEC) list, two reviewers independently assessed the risk of bias of the included economic evaluations. The list and the operationalisation of the criteria are described elsewhere [8]. Disagreements were discussed in a consensus meeting, and if necessary, a third reviewer (MvT) was consulted for a final decision.

### Data extraction

One reviewer (MTD) extracted the data from the included studies using a standardised data extraction form [9]. Information on study design, perspective of the economic evaluation, population, follow-up period, and measurements and valuations of costs and outcomes was extracted. Studies that expressed their costs in other currencies than the Euro were transformed to the Euro (exchange rate 30 March 2011). Publications (e.g., design papers or clinical outcomes paper) related to the included economic evaluations were used to gain extra information. A second reviewer (CCL) checked all data extracted. The primary outcome of the current review was the relative cost-effectiveness of the interventions, usually reported as an incremental cost-effectiveness ratio (ICER). The ICER indicates the additional monetary investments needed for the intervention to gain one extra unit of effect compared to the alternative treatment. In studies that found that one treatment was associated with lower costs and generated larger effects in comparison with the alternative treatment, the treatment is considered dominant, reporting of an ICER is not necessary. In this instance, if presented graphically, the ICER would be plotted in the south east quadrant of the cost-effectiveness plane [10].

For economic evaluations using quality-adjusted life years (QALY) to assess outcome, a cost-effectiveness threshold of the British National Institute for Health and Clinical Excellence [NICE; €22,000–€34,000 per QALY gained (£20,000–£30,000)] was used as an indicator of cost-effectiveness. That is, if a treatment resulted in an ICER lower than the NICE threshold when compared to an alternative, the treatment is considered to be relatively cost-effective [11, 12].

### Comparisons

For interpretation of the results we grouped the studies according to the following comparisons:

1. cost-effectiveness of manual therapy compared to other therapies
2. cost-effectiveness of physiotherapy compared to other therapies
3. cost-effectiveness of other therapies compared to any control.

## Results

### Study selection

**Fig. 1:**
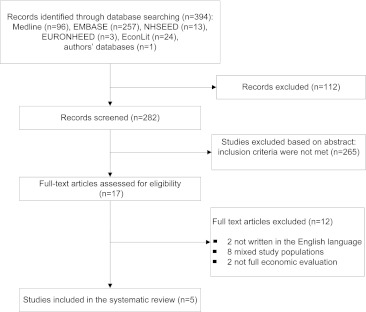
Flowchart of the included studies in this systematic review

The computer-generated search resulted in 282 titles and abstracts for screening. Most full papers were excluded, because, the study population reported on patients with non-specific low back pain and neck pain without separately presenting the effects and the costs for these two complaints. Other full-text articles were excluded, because they were not written in the English language or were not considered as a full economic evaluation. Altogether, five economic evaluations were included (Fig. 1).

### Study characteristics

Table 1 shows the characteristics of the included studies. Two studies were conducted in the United Kingdom, two in the Netherlands, and one in Germany. All studies conducted the economic evaluation from a societal perspective [13–17]; but two studies also addressed the health care perspective [15, 16]. All studies were randomised controlled trials with the number of participants ranging from 180 [13] to 3,451 [17]. Treatments were provided by professionals in primary health care (such as physiotherapists, manual therapists, acupuncturists, and general practitioners). The duration of non-specific neck pain among patients differed between studies from acute (2 weeks) [14, 16] to chronic (>12 weeks) [17]. Follow-up duration amongst studies varied from 3 [17] to 12 months [13, 14, 16]. While four studies conducted both cost-utility analyses (CUA) and cost-effectiveness analyses (CEA) [13–16], one study conducted CUA only [17]. Measures of clinical effects most often encompassed pain intensity, functional disability, and perceived patient recovery. Two studies measured pain intensity using an 11-point visual analogue scale [13, 14], and two studies assessed disability using the neck disability index [13, 15]. Two other studies assessed neck pain and disability using the Northwick Park neck pain Questionnaire (0–100 points and 0–36 points) [15, 16] and the neck pain and disability scale (5-point Likert scale) [17]. Studies focussing on perceived recovery used a 7-point [13] or a 6-point recovery scale [14]. Utilities were measured using the EQ-5D [14–16] or SF-6D [13, 17], then transformed into QALY. Index years for costs were specified in all but two studies [14, 17].

**Table 1:** Study characteristics

Study ID, type and perspective	Design	Groups	Effect differences (95 % CI)	Cost differences (95 % CI)	Incremental cost-effectiveness ratio
Korthals-de Bos et al. [14] Type: CEA/CUA Country: The Netherlands Perspective: societal Index year: not specified	Randomised controlled trial with a 12-month follow-up period. 183 patients with at least 2 weeks of non-specific neck pain.	(1) Manual therapy (MT): mobilisation and manipulation (one session per week up to six sessions) (2) Physiotherapy (PT): exercise, including relaxation exercises, stretching and functional exercises (30 min twice a week, up to 12 sessions) (3) General practitioner care (GP): standardised GP care + educational booklet (one session with optional fortnightly follow ups)	*Perceived recovery, % (6-point scale much worse –completely recovered)* MT vs. PT: 9.0 (−7.9 to 25.8) MT vs. GP: 15.4 (−1.3 to 32.1) PT vs. GP: 6.5 (−10.9 to 23.8) *Pain intensity (0–10 scale)* MT vs. PT: 1.2 (0.1–2.1) MT vs. GP: 0.1 (−0.8 to 1.1) PT vs. GP: −1.0 (−2.0 to 0.002) *Disability (Neck Disability Index 10 item 5-point scale)* MT vs. PT: 0.9 (−1.9 to 3.6) MT vs. GP: −1.4 (−4.1 to 1.3) PT vs. GP: −2.2 (−5.0 to 0.5) *QALY (EQ-5D)* MT vs. PT: 0.03 (−0.04 to 0.09) MT vs. GP: 0.05 (−0.01 to 0.11) PT vs. GP: 0.02 (−0.04 to 0.09)	MT vs. PT: €−850 (−2,258 to −239) MT vs. GP: €−932 (−1,932 to −283) PT vs. GP: €−82 (−1,063 to 1,446)	*Perceived recovery* MT vs. PT: €−9,488 MT vs. GP: €−6,041 PT vs. GP: €−1,265 *Pain intensity* MT vs. PT: €−757 MT vs. GP: €−6,652 PT vs. GP: €83 *Disability* MT vs. PT: €−967 MT vs. GP: €682 PT vs. GP: €36 *QALY* MT vs. PT: €−31,144 MT vs. GP: €−15,505 PT vs. GP: €2,688
Manca et al. [16] Type: CEA/CUA Country: United Kingdom Perspective: healthcare and societal Index year: 2001–2002	Randomised controlled trial with a 12-month follow-up period. 268 patients with at least 2 weeks neck pain.	(1) Brief intervention (BI): improve communication skills, de-medicalise the problem, and learn the principles of cognitive behavioural therapy (up to three sessions) (2) Usual PT (advice, MT, electrotherapy, acupuncture)	*QALY (EQ-5D)* BI vs. PT: −0.001 (−0.0030 to 0.028) *Pain (Neck Pain Questionnaire, 0–36 points)* BI vs. PT: 0.686 (−0.255 to 1.665)	*Healthcare costs* BI vs. PT: €−78 (−117 to −40) *Societal costs* BI vs. PT: €−117 (−331 to 99)	*Healthcare perspective* *QALY* €78,000 *Pain* €113 *Societal perspective* *QALY* €117,000 *Pain* €171
Willich et al. (2006) [17] Type: CEA/CUA Country: Germany Perspective: societal Index year: not specified	Randomised controlled trial with a 3-month follow-up period. 3,451 patients with chronic neck pain.	(1) Immediate acupuncture (10–15 sessions) (2) Delayed acupuncture after 3 months	*QALY (SF 6D)* Acupuncture vs. delayed acupuncture 0.024 (SE 0.004), *p* < 0.001	Acupuncture vs. delayed acupuncture € 293.91 (SE 51.79)	*QALY* €12,469
Lewis et al. [15] Type: CEA/CUA Country: United Kingdom Perspective: healthcare and societal Index year: 2003–2004	Randomised controlled trial with a 6-month follow-up. 350 patients with non-specific neck pain.	Interventions delivered over 6 weeks, with a maximum of eight 20-min sessions. (1) Advice and exercise: Individualised education, advice about coping with neck pain and a home exercise program (2) Advice and exercise + MT: passive and active assisted movements, mobilisations, and manipulations (3) Advice and exercise + pulse shortwave diathermy	*Neck pain and disability (Neck Pain Questionnaire 0–100 points)* Advice and exercise + MT vs. advice and exercise: −1.0 (−5.1 to 3.1) Advice and exercise + PSD vs. advice and exercise: −1.7 (−5.4 to 2.4) *QALY (EQ-5D)* Advice and exercise + MT vs. advice and exercise: 0.002 (−0.023 to 0.026) Advice and exercise + PSD vs. advice and exercise: 0.001 (−0.023 to 0.026)	*Healthcare costs* Advice and exercise + MT vs. advice and exercise: €6.8 (−39.03 to 44.28) Advice and exercise + PSD vs. advice and exercise: €20.5 (−22.94 to 55.21) *Societal costs* Advice and exercise + MT vs. advice and exercise: €−87.03 (−512.52 to 584.28) Advice and exercise + PSD vs. advice and exercise: €8.65 (−372.18 to 380.38)	*Healthcare perspective* *Neck pain and disability* Advice and exercise + MT vs. advice and exercise: €−6.80 Advice and exercise + PSD vs. advice and exercise: €−12.06 *QALY* Advice and exercise + MT vs. advice and exercise: €3,402 Advice and exercise + PSD vs. advice and exercise: €20,497 *Societal perspective* *Neck pain and disability* Advice and exercise + MT vs. advice and exercise: €87.03 Advice and exercise + PSD vs. advice and exercise: €−5.08 *QALY* Advice and exercise + MT vs. advice and exercise: €−43,513 Advice and exercise + PSD vs. advice and exercise: €8,652
Bosmans et al. [13] Type: CEA/CUA Country: The Netherlands Perspective: societal Index year: 2004	Randomised controlled trial with a 12-month follow-up period. 180 patients with sub acute (4–12 weeks) non-specific neck pain.	(1) Manual therapy: Manipulation and mobilisation (up to six 30 to 45-min sessions in 6 weeks) (2) Behavioural graded activity programme (BGA)	*Global perceived recovery (7-point scale completely recovered to worse than ever)* BGA vs. MT: 0.02 (−0.12 to 0.16) *Pain (0–10 scale)* BGA vs. MT: −2.4 (−4.5 to −0.22) *Disability (Neck Disability Index 10-items 5-point scale)* BGA vs. MT: −0.88 (−1.7 to −0.02) *QALY (SF-6D)* BGA vs. MT: −0.02 (−0.06 to 0.02)	BGA vs. MT €260 (−107 to 825)	*Global perceived recovery* €13,083 *Pain* €296 *Disability* €−296 *QALY* €−13,000

*CEA* cost-effectiveness, *CUA* cost-utility analyses, *MT* manual therapy, *GP* general practitioner, *PT* physiotherapy, *PSD* pulse shortwave diathermy, *BGA* behavioural graded activity, *CI* confidential intervals, *SE* standard error, *ICER* incremental cost-effectiveness ratio, *QALY* quality-adjusted life years

### Risk of bias

Table 2 shows the risk of bias assessment scores for the included studies. Because no study used a follow-up duration longer than 12 months, discounting was not needed. Therefore, it was decided to judge criterion number 14 (discounting) as not applicable. Two studies conducted sensitivity analyses to account for uncertainties in their costs and effects estimates. Three studies did no conduct a sensitivity analysis. Costs were most often assessed by using cost diaries or questionnaires [13–16], and studies appropriately valued the obtained costs according to published sources. All studies provided an incremental costs-effectiveness analysis, and four studies presented cost-effectiveness planes [13–15, 17].

**Table 2:** Risk of bias assessment scores

Criterion	Korthals-de Bos et al. [14]	Manca et al. [16]	Willich et al. [17]	Lewis et al. [15]	Bosmans et al. [13]
Study population	+	+	+	–	+
Competing alternatives	+	+	+	+	+
Research question	+	+	+	+	+
Study design	+	+	+	+	+
Time horizon	+	+	–	–	+
Perspective	+	+	+	+	+
Relevant costs	+	+	–	+	+
Physical units	+	+	–	+	+
Appropriate valuation	+	+	–	+	+
Relevant outcomes	+	+	+	+	+
Outcomes appropriately measured	+	+	+	+	+
Appropriate outcome valuation	+	+	+	+	+
Incremental analyses	+	+	+	+	+
Discounting	N/A	N/A	N/A	N/A	N/A
Sensitivity analyses	–	–	+	+	–
Adequate conclusions	+	+	+	+	+
Generalisability	–	+	+	+	+
Conflict interests	+	+	+	+	+
Ethics discussed	+	+	+	+	+

**+,** adequately reported/low risk of bias; **−,** not adequately reported/high risk of bias; N/A, criterion is not appropriate

### Cost-effectiveness of manual therapy

#### Manual therapy compared to physiotherapy

One study (*n* = 183) conducted in the Netherlands compared manual therapy (consisting of spinal mobilisation and manipulation) with physiotherapy (consisting of functional exercises, relaxation, and stretching) among patients with at least 2 weeks of non-specific neck pain [14]. After 12 months, the results showed that manual therapy was statistically significantly more effective in reducing pain intensity but not on perceived recovery or QALY. However, the manual therapy group was accompanied with lower costs, resulting in an ICER of €−757 per point pain intensity reduction and €−9,448 per percentage recovered. The costs-effectiveness planes showed that for pain intensity 98 %, for perceived recovery 85 % and for QALY 87 % of the bootstrapped ratios were located in the southeast quadrant, indicating that manual therapy is dominant over physiotherapy.

#### Manual therapy compared to general practitioner care

Korthals-de Bos et al. [14] (*n* = 183) also compared manual therapy with general practitioner care (standardised guideline care). The costs-effectiveness planes showed that, respectively, 96 % for perceived recovery and 87 % for QALY of the bootstrapped ratios were located in the southeast quadrant, indicating that manual therapy is dominant over general practitioner care. No differences in cost-effectiveness between manual therapy and general practitioner care were found on pain intensity and disability.

#### Manual therapy plus advice and exercise compared to advice and exercise

The study of Lewis et al. [15] (*n* = 350) compared advice and exercise with advice, exercise and manual therapy. The cost-effectiveness planes showed that there is no difference in cost-effectiveness between the two interventions. At the €34,000 per QALY threshold the probability of manual therapy to be cost-effective was 0.37 from the health care perspective and 0.44 from the societal perspective.

#### Manual therapy compared to behavioural graded activity programme

One study (*n* = 180) compared manual therapy (manipulation and mobilisation) with a behavioural graded activity programme (time contingent exercise programme) amongst patients with sub acute neck pain [13]. The cost-effectiveness planes showed that behavioural graded activity programme was not cost-effective.

### Cost-effectiveness of physiotherapy

#### Physiotherapy compared to manual therapy

See paragraph on “Manual therapy compared to physiotherapy” above.

#### Physiotherapy compared to general practitioner care

One study (*n* = 183) compared physiotherapy with general practitioner care amongst patients who had at least 2 weeks of non-specific neck pain [14]. The cost-effectiveness planes showed that there is no difference in cost-effectiveness between the two treatment options.

#### Physiotherapy compared to brief physiotherapy intervention

One study (*n* = 268) compared a brief physiotherapy intervention (encouragement of return to normal activities using cognitive behavioural principles) with usual physiotherapy (including electrotherapy, manual therapy, advice, exercise and acupuncture) amongst patients who had for at least 2-week neck pain [16]. Regarding QALY it was found that from both the health care and societal perspective, the brief physiotherapy was not cost-effective when compared with usual physiotherapy.

### Cost-effectiveness of other therapies

#### Advice and exercise compared to advice and exercise with pulsed shortwave diathermy

The study of Lewis et al. [15] (*n* = 350) compared advice and exercise to advice and exercise with pulsed shortwave diathermy. The cost-effectiveness planes showed that there was no difference in cost-effectiveness between the two interventions. At the €34,000 per QALY threshold, the probability of pulsed shortwave diathermy to be cost-effective was 31 % from the health care perspective and 26 % from the societal perspective.

#### Acupuncture compared to delayed acupuncture

Amongst 3,451 chronic neck-pain patients, Willich et al. [17] compared the cost-effectiveness of acupuncture versus delayed acupuncture. The follow-up duration was short (3 months). All bootstrapped ratios were located in the northeast quadrant of the cost-effectiveness plane. The ICER of €12,469 per QALY gained indicates that acupuncture in comparison with delayed acupuncture is cost-effective for the treatment of chronic neck pain.

## Discussion

The aim of this systematic review was to investigate the cost-effectiveness of conservative treatment options for non-specific neck pain. The number of economic evaluations included was limited, and pooling of data was not possible due to heterogeneity between the studies. Therefore, drawing firm conclusions from the included economic evaluations is not possible. Nonetheless, the systematic review revealed several important discussion points and gained insight into items that may improve the reporting of future economic evaluations on neck pain.

One economic evaluation conducted in the Netherlands found that manual therapy was the dominant and cost-effective treatment option when compared to physiotherapy and general practitioner care [14]. Another study conducted in the Netherlands, found that behavioural graded activity was not cost-effective over manual therapy [13] whereas a study conducted in the UK found that manual therapy combined with advice and exercise was not cost-effective in comparison with advice and exercise [15]. Comparison of these study findings is difficult because of the different socio-political context (compensation systems, insurance systems, and jurisdictions) and the different health-care systems across countries [10, 18]. Moreover, heterogeneity of the study population also hampers comparison. Korthals-de Bos et al. [14] and Lewis et al. [15] treated patients with at least 2 weeks of neck pain. As a consequence, study groups consisted of a mix of patients with acute, sub acute, or chronic non-specific neck pain. In contrast, Bosmans et al. [13] included only patients with sub acute non-specific neck pain. In addition, the manual therapy studies differed in control group(s) used, follow-up duration (6 or 12 months), and the treatment provider of the manual therapy (manual therapist [13, 14] and regular physiotherapist [15]). Regarding the other studies, heterogeneity existed on the assessment of the outcome measures (disability measured by NDI, NPAD or NPQ) and costs (diaries or databases), participants (mix of acute, sub acute, and chronic), interventions (brief physiotherapy intervention, manual therapy, acupuncture), controls (delayed intervention, usual physiotherapy, general practitioner care), and follow-up duration (3, 6, 12 months). Consequently, it was impossible to statistically pool the data.

Similar to low back pain, the cost for neck pain due to production loss and sick leave are almost nine times higher compared to the health care costs [19]. A gold standard for collecting sick leave data is not available yet. The included studies used either questionnaires or data from the insurance company to measure the productivity loss and sick leave. A disadvantage of insurance databases is that they provide information on the amount of money that has been compensated, but does not provide information about the actual time someone was not at work. Self-reports have the disadvantage that they may be unreliable due to recall bias. Therefore, the estimated costs as reported in the included economic evaluations may be over- or underestimations of the actual costs. Furthermore, productivity changes and changes in work performance due to sick leave are most often not incorporated in the sick leave costs, and may thereby further underestimate the costs [18].

Most of the economic evaluations included in this systematic review conducted both cost-effectiveness and cost-utility analyses. The information obtained from the cost-effectiveness analyses is limited, because, they only provide relevant information for clinicians (i.e., €600 per point pain-intensity reduction). For policy makes, however, results of the cost-utility analyses are better interpretable, because, a generic outcome such as the QALY is used which can be compared across interventions and health conditions [20]. We used the NICE cut-off to determine the cost-effectiveness neck pain treatments. Although the use and the height of such a cut-off point is under debate, most of the cost-effective treatments for neck pain studied in the current review were below the €34,000 threshold [21, 22].

Irrespective of the results, presenting an ICER and a cost-effectiveness plane in an economic evaluation is important. This is because ICERs do not provide any information about the uncertainty around the cost-effective estimate. All of the studies included in this review presented the ICER, and, with the exception of the study of Manca et al. [16], all studies presented a cost-effectiveness plane. To improve the interpretation of the cost-effectiveness of an intervention we support that economic evaluations always present both ICERs and cost-effectiveness planes.

The current systematic review only included five economic evaluations on the (conservative) treatment of non-specific neck pain, which is significantly less than the number of randomised controlled trials that have been conducted in this field [6, 7]. These economic evaluations were performed in the Netherlands (*n* = 2) [13, 14], the UK (*n* = 2) [15, 16], and Germany (*n* = 1) [17]. Whereas randomised controlled trials investigating the effectiveness of conservative treatments for neck pain have been conducted in the United States [23, 24], Australia [25], Scandinavia [26] and Canada [27], economic evaluations on these studies were not found.

## Conclusion

The results indicate that manual therapy is more cost-effective than physiotherapy or GP care. Acupuncture is also cost-effective in the short term, but adding treatments to advice and exercise is unlikely to be cost- effective. These findings were obtained from single studies only. The small number of economic evaluations for treatments of non-specific low back-pain limits firm conclusions to be made on their cost-effectiveness.

## Appendix: Medline search

#1 Study design:

“Cost-Benefit Analysis” [MeSH] OR “economic evaluation” [tiab] OR cost effectiveness [tiab] OR “economic analysis”[tiab] OR cost effective*[tiab] OR (cost[tiab] OR costs[tiab] AND (benefit*[tiab] OR utilit*[tiab] OR effective*[tiab] OR minimisation[tiab] OR minimisation[tiab])).

#2 Neck pain:

“Neck Pain” [MeSH] OR “Neck pain” [tiab] OR “neck ache” [tiab] OR “neck aches” [tiab] OR “neck complaints” [tiab] OR “neck injury” [tiab] OR “neck symptoms” [tiab] OR “neck injury” [tiab] OR “upper limb symptoms” [tiab].
